# Dynamics of oil–water interface at the beginning of the ultrasonic emulsification process

**DOI:** 10.1016/j.ultsonch.2023.106657

**Published:** 2023-10-21

**Authors:** Žan Boček, Martin Petkovšek, Samuel J. Clark, Kamel Fezzaa, Matevž Dular

**Affiliations:** a Faculty of Mechanical Engineering, University of Ljubljana, Askerceva 6, 1000 Ljubljana, Slovenia; b Advanced Photon Source, Argonne National Laboratory, 9700 S Cass Ave, Lemont, IL 6043, USA

**Keywords:** Cavitation, Emulsion, High speed video, X ray visualization

## Abstract

A lot of effort has been dedicated in recent years towards understanding the basics of cavitation induced emulsification, mainly in the form of single cavitation bubbles. Regarding bulk acoustic emulsification, a lot less research has been done. In our here presented work we utilize advanced high-speed observation techniques in visible light and X-Rays to build upon that knowledge and advance the understanding of bulk emulsion preparation. During research we discovered that emulsion formation has an acute impact on the behavior of the interface and more importantly on its position relative to the horn, hence their interdependence must be carefully studied. We did this by observing bulk emulsification with 2 cameras simultaneously and corroborating these measurements with observation under X-Rays. Since the ultrasonic horns location also influences interface behavior, we shifted its initial position to different locations nearer to and further away from the oil–water interface in both phases. We found that a few millimeters distance between the horn and interface is not enough for fine emulsion formation, but that they must be completely adjacent to each other, with the horn being located inside the oil–water interface. We also observed some previously undiscovered phenomena, such as the splitting of the interface to preserve continuous emulsion formation, climbing of the interface up the horn and circular interface protrusions towards the horn forming vertical emulsion streams. Interestingly, no visible W/O emulsion was ever formed during our experiments, only O/W regardless of initial horn position.

## Highlights

• We show precise observations of initial stages of ultrasonic emulsification.
• High-speed observation techniques in visible light and X-Rays were used.
• Regardless of the initial setup, only oil in water emulsion forms.
• For efficient emulsification the tip needs to be in close vicinity to the interface.

## Graphical abstract

**Figure:**
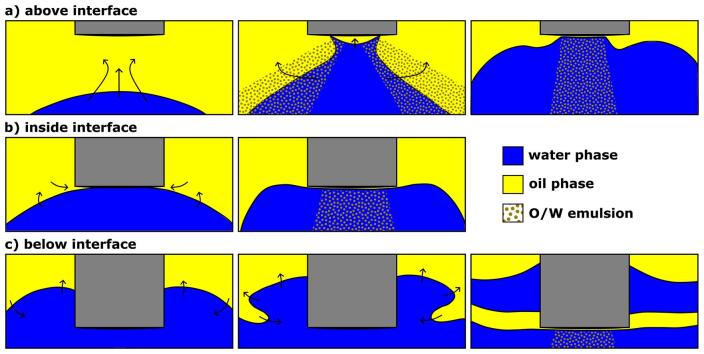

## 1 Introduction

Emulsions are heterogeneous systems prominently used in food and beverage industry, as well as in medical, cosmetic, agricultural, and pharmaceutical compounds [1], [2], [3]. They are composed of 2 immiscible liquids, where one is dispersed into the other [4], [5]. Hence, they can be classified as oil in water (O/W) or water in oil (W/O) emulsions and more complexly as oil in water in oil (O/W/O) or water in oil in water (W/O/W) emulsions. Their kinetic stability favors higher interfacial areas (smaller droplets) [6].

Since the appearance and functionality of emulsions are greatly influenced by their quality and as such by their preparation, considerable care must be put into it [2]. For an emulsion to achieve a metastable state, extreme amounts of energy must be applied during production [1], [6], [7]. This emulsification energy can be supplied either mechanically (by high-pressure homogenizers, static mixing, rotor–stator systems etc.) or through ultrasound [5]. The latter produces a more homogenous emulsion while requiring less energy, maintenance, smaller amounts of surfactant and lower costs [6], [8]. However, researchers focusing on detailed applications of ultrasound emulsification has left the physical phenomena behind them largely unexplained [2].

Ultrasound is generated by the oscillation of a piezoelectric material and transmitted into a liquid. There it generates acoustic streaming and mechanical vibrations, which causes the gas nuclei inside the liquid to grow into bubbles, oscillate and collapse, thus creating acoustic cavitation [9]. The collapsing bubbles microstreaming and shockwaves greatly improve mass transfer and fluid mixing – the so-called mechanical effects of acoustic cavitation [2].

The current understanding of ultrasound emulsification describes it as a complex sequence of phenomena caused by transient cavitation [10], [11]. Perdih et al. [10] demonstrated that during bulk emulsification firstly a W/O or O/W/O emulsion forms inside the bulk oil phase, later separating from it into the water phase, where it breaks down into an O/W emulsion. This dispersion is believed to be caused by liquid jets and shockwaves of collapsing bubbles. The collapsing bubble jets away from the oil–water interface if the cavitation bubble grows inside the denser phase (i.e., the water phase) and jets towards it if it grows inside the lighter phase (i.e., the oil phase) [12].

The renewed interest in the fundamentals of cavitation emulsification mechanisms has been stirred up and amended by Perdih et al. [10]. Building on their gained insight, Orthaber et al. [12] in 2020 undertook further research of individual cavitation bubbles near the interface between a single oil droplet and a bulk water phase, where they identified the direction of bubble jetting regarding cavitation location. Furthermore, have Orthaber et al. [13] in another paper explored cavitation emulsification interactions in the vicinity of a flat liquid–liquid interface, again on the scale of single bubbles. Raman et al. [14] investigated single cavitation bubble induced emulsification of different viscosity silicone oils with a water droplet, whose regimes and boundary conditions they expect to be applicable to ultrasound emulsification. They identified 3 interaction regimes dependent on oil viscosity, maximum bubble diameter and distance between droplet and bubble center: deformation, external emulsification and internal emulsification. Research of ultrasound emulsification with gas bubbles and bulk liquids was also conducted by Wu et al. [15] in 2021, but observations of practical ultrasonic horn emulsification beyond single cavitation bubbles have, to the best of our knowledge, not been undertaken to the extent and detail as presented in this paper.

Tiong et al. [16] focused more on the numerical side of bulk acoustic emulsification, with very basic experimental observations. They found that areas where the acoustic pressure exceeds the cavitation threshold were larger if the horn was placed closer to the oil–water interface, producing smaller emulsion droplets. Cucheval and Chow [5] established in 2008 that an emulsion cloud only forms if the oil–water interface is located a few millimeters below the horn, where transient cavitation is present. For these types of complex systems and for further process optimization the proposed distance of a few millimeters is too broad. They encountered some limitations during their observations, namely a too low framerate and no detailed observations of the events directly below the horn. The thin layer of oil used didn’t account for interface movement caused by bulk emulsion production, which influences the emulsification process itself. Prior research also didn’t consider the horns presence and location on emulsification mechanisms. With our work we amend the understanding of bulk acoustic emulsification by more detailed observations and by carefully studying the interdependence of the ultrasonic horn and oil–water interface.

In the present paper we first describe the setups for visualization in visible and X-Ray wavelengths, which enabled detailed observation of emulsification of two liquids in a bulk. This brings the investigation to more application-level conditions, which is many times omitted due to complexity of experiments. The influence of the submergence of the ultrasonic horn and the distance between ultrasonic horn and interface is then extensively presented through different examples, followed by a discussion of various mechanisms and phenomena behind emulsification.

## 2 Experimental set-up

### 2.1 Materials

Emulsions were prepared with distilled water and a mineral hydraulic oil with a density of 869 kg/m^3^ and kinematic viscosity of 46 mm^2^/s. The ultrasonic horn used was a Cole-Parmer 750-Watt Ultrasonic with a frequency of 20 kHz, equipped with a 3.2 mm titanium microtip and set to 20 % of its maximum amplitude, which corresponds to 137 μm peak to peak amplitude.

The measurements of ultrasonic horn emulsification were performed in standard 4 mL plastic cuvettes with 10 × 10 × 45 mm inner dimensions. They were filled with fresh 2.5 mL of distilled water and 1.0 mL of oil for each experiment. Attention was paid to cleaning the ultrasonic horn’s tip between experiments to prevent premature emulsion formation from the remains of previous experiments. This way the only location of emulsion formation would be at the phase boundary between bulk layers.

### 2.2 Measurements under visible light

**Fig. 1:**
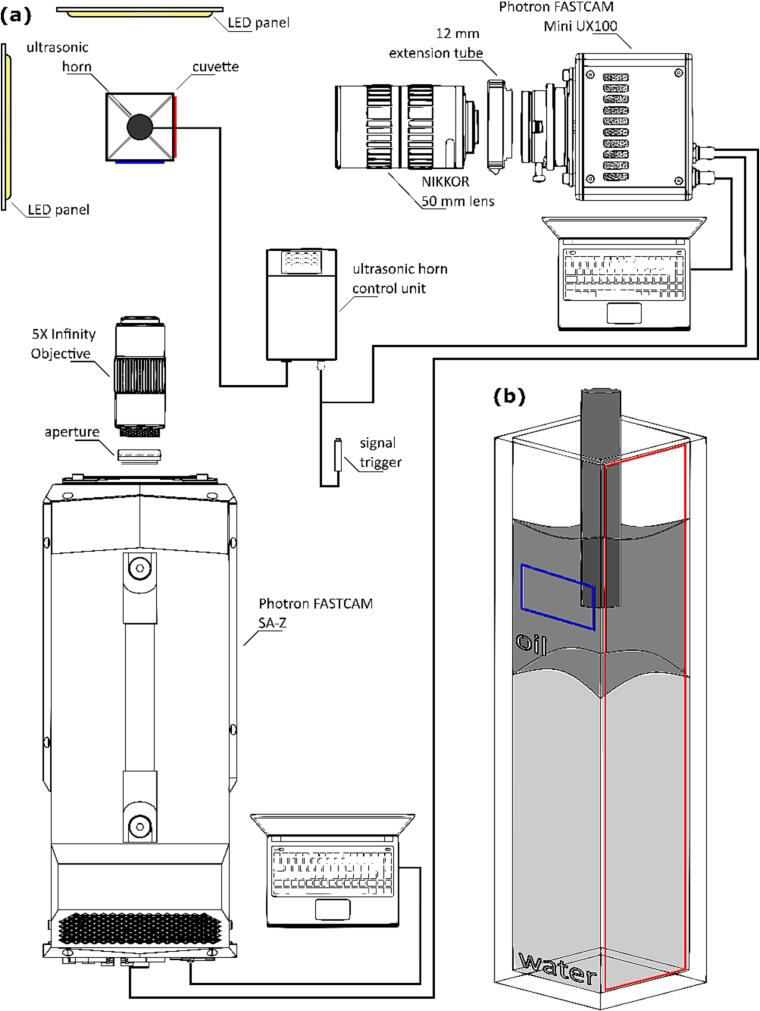
Experimental set-up for observations under visible light: (a) lens configuration and equipment position, (b) individual cameras field of view (blue – Photron SA-Z, red – Photron Mini UX100). (For interpretation of the references to colour in this figure legend, the reader is referred to the web version of this article.)

Emulsification observations were done simultaneously with 2 high-speed cameras at an angle of 90° (Fig. 1a):

• for the detailed view Photron FASTCAM SA-Z, set to a resolution of 384 × 160 pixels, a frame rate of 210 000 frames per second and a shutter speed of 3.15 µs was equipped with an aperture and a 5X Mitutoyo Plan Apo Infinity Corrected Long WD Objective;
• for the integral view Photron FASTCAM Mini UX100, set to a resolution of 1280 × 248 pixels, a frame rate of 20 000 frames per second and a shutter speed of 50 µs was equipped with a 12 mm macro extension tube and a NIKKOR 50 mm lens.

The resulting fields of view were 8.5 mm × 3.5 mm and 10 mm × 45 mm for SA-Z and Mini UX100 respectively (Fig. 1b).

Back light illumination of the cuvette, ultrasonic horn microtip and sample was provided by 2 LED panels (one for each camera). Camera recording and ultrasonic horn activation was triggered manually, through a BNC cable splitter simultaneously connected to the ultrasonic horn and Photron Mini UX100. The Mini UX100 camera then passed the trigger signal forward to the Photron SA-Z.

### 2.3 Measurements under x-rays

High speed X-Ray measurements were conducted at the Argonne National Laboratory – Advanced photon source (Sector 32-ID-B). Images were captured by a Phantom TMX 6410 high-speed camera with a 10x magnification through a LuAG:Ce scintillator screen with a field of view of 1.5 mm × 0.5 mm. The camera’s resolution was set to 1280 × 416 pixels and synchronized with the hybrid APS timing mode. The hybrid timing mode consists of a single bunch isolated from a super-bunch consisting of 8 groups of 7 consecutive bunches. The camera was synchronized with every other super-bunch (approx. 135.5 kHz) giving an effective exposure time of 500 ns and eliminating motion blur. The 33 mm long period undulator at 32-ID-B which provides a highly intense X-ray beam with a broad energy spectrum from 7 to 40 keV.

**Fig. 2:**
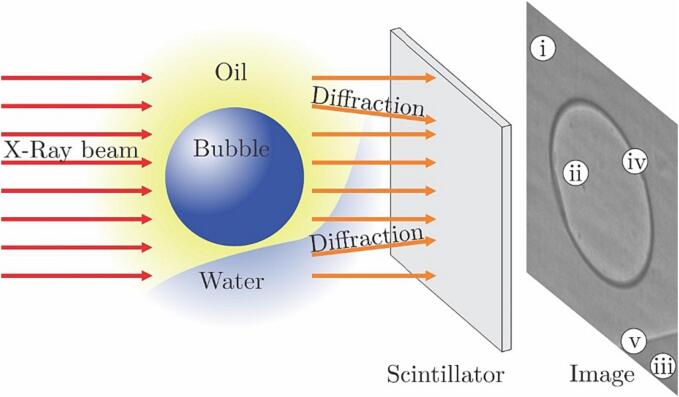
The principle of X-Ray detection and interpretation of 2 phase flow images.

The phase contrast imaging technique, used in the present experiment, relies mainly on the interference between the X-Rays, which are diffracted at the liquid–vapor or liquid–liquid interfaces and the non-difracted ones (Fig. 2).

In addition, the light attenuation is related to the material properties the beam passes on its way to the detector – vapor has the smallest attenuation coefficient, oil has a much larger one and water has the highest; hence the bubble will appear the brightest, while oil and water will be darker in the image. To achieve the interference the beam must be (at least partially) spatially coherent, the condition which can be easily satisfied by a third-generation synchrotron like the one of the Argonne National Laboratory. The beam is projected onto a scintillator screen, which converts the X-Ray to visible light that is recorded by the high-speed camera.

The final image (a typical single bubble image is shown in Fig. 2) consists of five regions which can be identified as: i) liquid oil (low intensity due to high attenuation), ii) vapor (high intensity due to low attenuation), iii) liquid water (very low intensity due to highest attenuation), iv) liquid oil/vapor interface (low intensity due to diffraction) and v) liquid oil/liquid water interface (low intensity due to diffraction).

The technique allows for imaging throughout the depth of the cuvette, revealing details that are unseen in classical backlight high-speed imaging.

### 2.4 Emulsion size analysis

Emulsion droplet size analysis was conducted with an optical microscope (Zeiss Axio Observer Z1, equipped with laser confocal unit LSM 800). 10 µL of prepared emulsion was transferred onto a microscopic glass slide and covered with 20 × 20 mm #1.5 cover glass. The edges of the #1.5 cover glass were sealed with silicone grease to prevent emulsion droplet movement due to capillary forces. Samples were observed with a 60x magnification, resulting in a 189.1 × 189.1 µm observation area with a resolution of 2048 × 2048 pixels. For each sample, microscopic images were acquired in 3 random locations in a 6 × 6 tiled grid with 10 µm overlap. Each experiments was repeated 5 times (resulting in roughly 500 images for each position of the ultrasonic horn). Image analysis was conducted through ImageJ (1.53 t) and Python (3.10.9) script utilizing Hough Circle Transformation. The Python script was written to not take into account droplets smaller than 1 µm and larger than 10 µm to prevent image cluttering. The diameters of the detected circular objects i.e., emulsion droplets, were used to calculate the droplets’ relative size distribution and relative area of solution covered by emulsion droplets.

## 3 Results

In the present study we focused on the specifics of the horn and cavitation interaction with the oil–water interface. Hence different distances of the tip of the horn from the initial interface were investigated. These were 2 and 1 mm above the interface, 1 and 2 mm submerged below the interface and additional three in a close vicinity of the interface – just above, just below and within the interface. For each condition, the experiment was repeated at least 5 times, in both visual and X-Ray spectrum. The general mechanisms and sequence of events were the same within 5 parallels, they did however differ in time intervals between individual steps. As such, the examples provided and discussed in individual sections were chosen based on greatest clarity of observed events. In addition, during individual experiments some atypical occurrences were observed - these are addressed separately.

For observations made in visible light the timestamps for every consequent noteworthy event are presented in milliseconds from the point of the first movement of the ultrasonic horn t_0_. In figures showing the synchrotron measurements, the t_0_ frames were chosen for the first interesting event.

For each case the integral view is shown (recorded by Photron Mini UX100 camera) and a closeup perpendicular view (recorded by Photron SA-Z camera) are shown (see also Fig. 1b).

Since the focus of the present study was to observe the emulsification process the term “interface” refers to the phase boundary between the oil and the water phase (unless specified otherwise).

### 3.1 Ultrasonic horn positioned above the oil–water interface

To investigate the behavior of the oil–water interface during emulsification mechanisms, we firstly positioned the ultrasonic horn 2.0 mm and 1.0 mm above the uppermost layer of said interface. The discussion is mainly based on observations in visual light, these are complemented by X-Ray measurements.

#### 3.1.1 2.0 mm above the interface

**Fig. 3:**
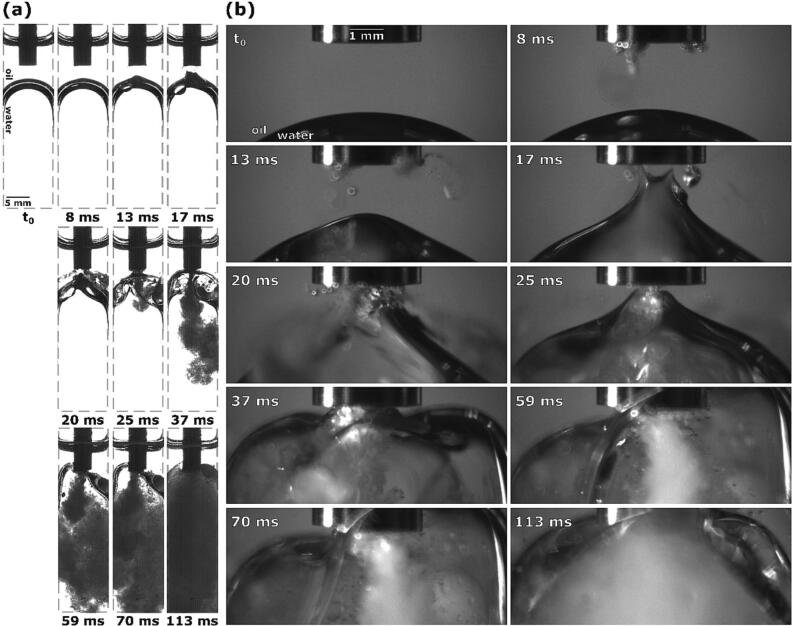
Sequence of events during ultrasonic emulsification under visible light with the ultrasonic horn positioned 2.0 mm above the oil–water interface: (a) observation with Photron Mini UX100, (b) observations with Photron SA-Z.

Looking at Fig. 3 we see that 8 ms after the ultrasonic horn activates, two cavitation areas form underneath the tip: near the left and the right corner (better seen in closeup image Fig. 3b). A close inspection of the second image in Fig. 3b also reveals appearance of an emulsion cloud, which starts to expand from the left cavitation area (marked with a red dashed line). Neither the initial cavitation nor the emerging emulsion is visible Fig. 3a. Here we can clearly observe the beginning of interface deformation below the horn’s left corner, caused by the ever-growing emulsion cloud.

Both cavitation areas underneath the ultrasonic horn continue to grow and the emulsion cloud from the left cavitation area continues to expand, which results in the interface’s more extensive downward indentation. Additionally, 13 ms after horn activation, a new emulsion cloud starts to form and spiral clockwise out from the right cavitation area, evident by the “tail” that appears. Due to emerging currents, the interface is being pulled towards the horn, forming a cone. This cone shape is also noticeable in Fig. 3a, as well as a thin layer of cavitation below the horn’s tip.

Cavitation in both areas continues to expand. The cone from the interface’s top layer protrudes higher with time, at 17 ms reaching the horn itself. During this, either 1 or in some cases 2 peaks emerge from the aforementioned interface cone, curving outwards in accordance with circular currents present at the horn’s perimeter [17]. The cone’s very peak is not visible in Fig. 3a, probably due to it being too thin and not able to obstruct enough light.

As more of the interface makes physical contact with the horn, and more importantly the cavitation beneath it, at 20 ms, fine emulsion streams start to form diagonally from both corners, spreading to the whole width of the cuvette.

25 ms after horn activation, the cone shaped protrusion from the upper interface layer becomes shorter and wider as the whole interface is being pushed upwards due to the formed emulsion adding to the water phase’s volume. This causes the 2 circular peaks (marked with dashed red lines) and consequently the diagonal emulsion streams to disappear, instead connecting a wider part of the interface to the horn, which in turn starts to form a vertical emulsion stream.

Additionally, the lower interface layer splits off (Fig. 3a at 25 ms), forming 2 separate segments that both curve up towards the ultrasonic horn and into the emerging emulsion stream. Further emulsion formation causes the top of the interface to move higher up and level out, causing it to curve below the tip’s corners. As more of the interface reaches the tip at 37 ms, emulsion formation slows down. The curvature of lower interface parts in the meantime becomes more distinct, being directed opposite to the emulsion flow, but in accordance with suspected circular currents at the horn’s corners [17].

As the interface moves further up and most of it reaches over the tip at 59 ms (Fig. 3b), emulsion formation again becomes more extensive. Simultaneously with the interface upwards movement, it splits into two parts and its lower section stays level with the horn’s tip, while the upper part continues to rise higher (Fig. 3a, left of the horn).

Emulsion continues to form with roughly the same extent and as such continues to increase the water phase’s volume, but since most of the water has already been replaced by emulsion up to this point, additional changes in overall interface height are negligible. However, some parts of the lower interface layer (Fig. 3a, left of the horn) split off from the horn’s tip at 70 ms. 113 ms after ultrasonic horn activation, the water phase is completely replaced by the formed emulsion (Fig. 3a) and hence the area around the horn’s tip becomes undiscernible (Fig. 3b).

**Fig. 4:**
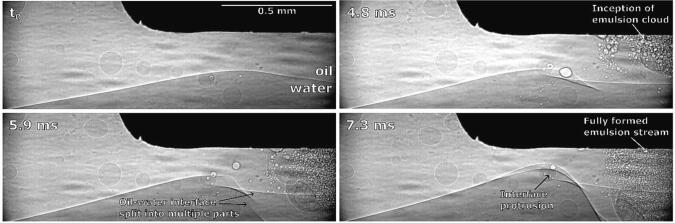
Synchrotron observations of interface indentation and interface peak formation below ultrasonic horn due to growing emulsion cloud.

The observations made in visible light can be further supported by synchrotron measurements (Fig. 4).

Fig. 4 shows a growing emulsion cloud under the center of the horn that causes the oil–water interface to split, part of it being pushed down and radially outwards, forming a peak under the perimeter of the horn – revealing details of the observation of interface indentation shown in Fig. 3a at 8 ms and 13 ms.

**Fig. 5:**
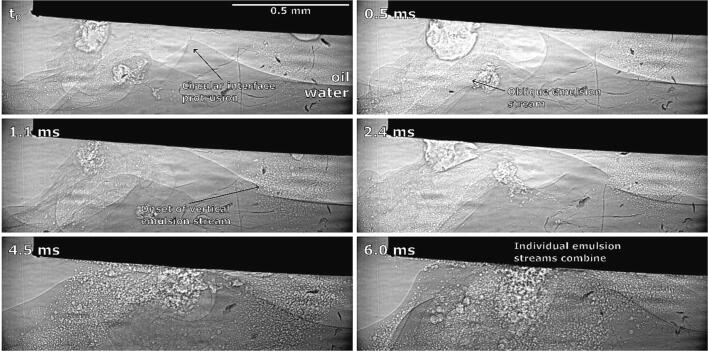
Synchrotron observations of interface peak rotation due to circular currents underneath corner of ultrasonic horn and subsequent emulsion formation.

In the following, details on some phenomena shown in Fig. 3 are further discussed. In Fig. 3b (at 17 ms) circular interface protrusion was shown. This can be explained by looking at Fig. 5, which shows the corresponding X-Ray measurements.

Note that a fresh ultrasonic horn tip was used (compared to the one in Fig. 4) - this does not change the dynamics of emulsification significantly. We can see that an initial emulsion cloud starts to form near the horn’s corner. Afterwards an interface peak rotating in the direction of circular flow field appears. It later vanishes as it connects with the horn. As a consequence, the intensity of the diagonal emulsion stream increases.

**Fig. 6:**
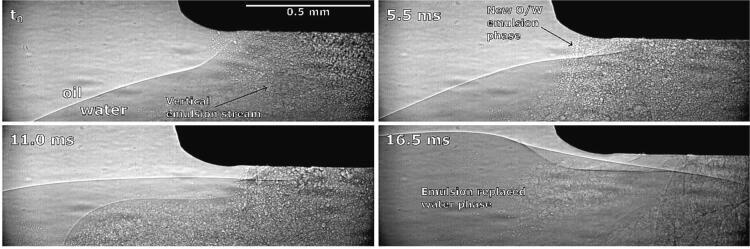
Synchrotron observation of vertical emulsion stream and oil–water interface movement around ultrasonic horn.

Fig. 6 shows the upwards movement of the interface and its curving around the horn’s corner (a phenomenon similar to the one shown in Fig. 3b at 37 ms).

Here the oil–water interface is directed towards the ultrasonic horn from below and a comparable vertical emulsion stream is present. The interface moves noticeably higher throughout emulsion production, splits into individual layers and ultimately rises above the horn’s tip, forming an indentation near the horn’s corner.

#### 3.1.2 1.0 mm above the interface

General emulsification mechanisms from these cases are comparable to those found in Section 3.1.1 where the distance between horn and interface was 2.0 mm.

**Fig. 7:**
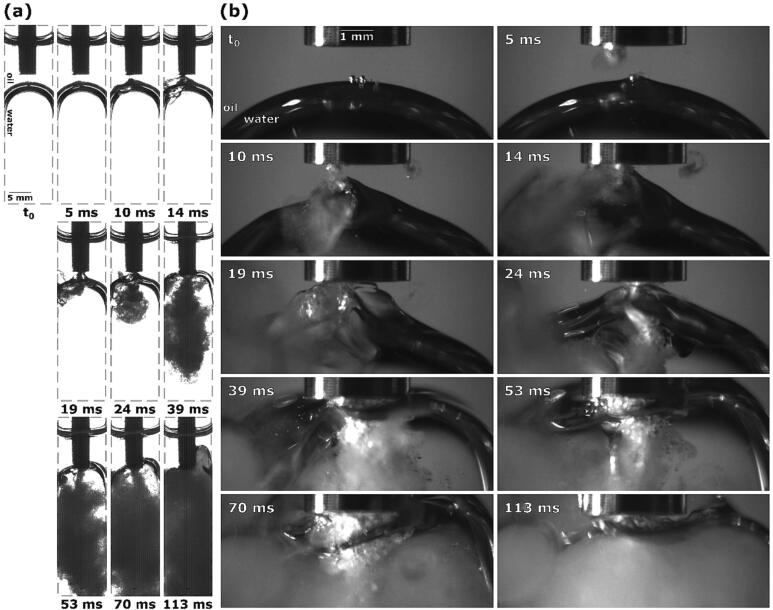
Sequence of events during ultrasonic emulsification under visible light with the ultrasonic horn positioned 1.0 mm above the oil–water interface: (a) observation with Photron Mini UX100, (b) observations with Photron SA-Z. Individual phenomena are similar to those presented on Fig. 3, mostly differing in time of appearance and appearing sooner due to a closer distance between horn and interface.

When the ultrasonic horn lies lower, some phenomena appear sooner due to its shorter distance to the interface. For example, the interface peak generally starts to form earlier and simultaneously with the cavitation underneath the horn (Fig. 7b at 5 ms). Also, both cavitation/emulsion clouds near the horn’s corners are less likely to appear at the same time, which means that the growing interface peak will preferentially incline towards either side (Fig. 7b at 10 ms). However, a comparable diagonal emulsion stream will still form (Fig. 7a at 14 ms).

The vertical emulsion stream also starts to form sooner when the horn is positioned closer to the oil–water interface, although the difference only amounts to a few milliseconds. As the emulsion formation approaches its ending, differences in timing between examples become smaller, at the end completely disappearing.

### 3.2 Ultrasonic horn in the vicinity of oil–water interface

For the investigation of emulsion formation behavior when the oil–water interface is in immediate vicinity of the ultrasonic horn, 3 sets of experiments were conducted, with the ultrasonic horn’s tip placed directly above, inside, and directly below the oil–water interface.

#### 3.2.1 Directly above the interface

**Fig. 8:**
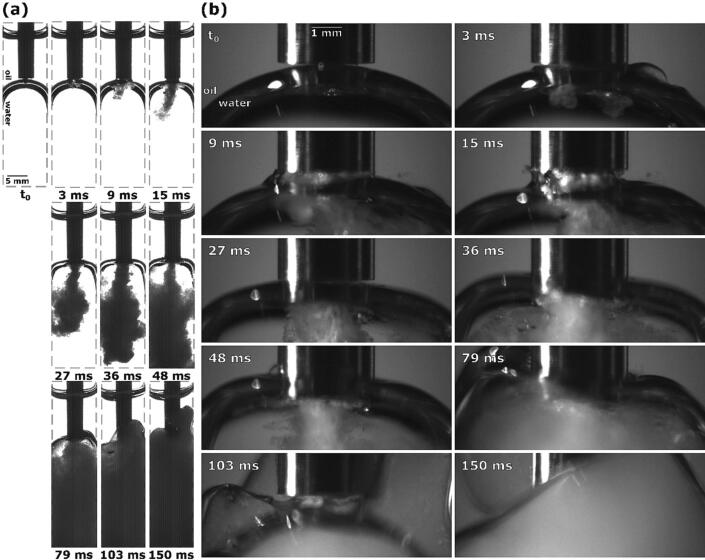
Sequence of events during ultrasonic emulsification under visible light with the ultrasonic horn positioned directly above the oil–water interface: (a) observation with Photron Mini UX100, (b) observations with Photron SA-Z.

Firstly, we show the case when the tip was placed directly above the interface, slightly touching its upper layer (Fig. 8 at t_0_).

3 ms after horn activation, cavitation begins to appear. Simultaneously, the nearest interface segments are pulled up towards the horn (Fig. 8b), increasing their contact surface. This enables initial emulsion formation in the form of two circular emulsion clouds at the horn’s corners inside the interface (Fig. 8a).

Oil-water interface continues to be pulled upwards, at 9 ms spanning the whole width of the horn and forming a “neck” around it. Also currently, 2 individual emulsion clouds combine into a downwards stream of emulsion.

The emerging emulsion stream causes the interface to be pushed downwards, increasing the distance between tip and interface, in turn narrowing the aforementioned interface “neck” and curving it inwards on the sides. At 15 ms the emulsion stream becomes stronger (Fig. 8a).

As the intensity of the emulsion stream increases even further (27 ms after activation), the interface becomes slightly narrower and noticeably flatter, most likely due to the formed emulsion increasing the water phase’s volume, starting to push the whole interface upwards. A noticeable amount of emulsion has formed inside the water phase up to this point.

36 ms after initial horn activation, the emulsion stream again increases in intensity, visible by its increased width (Fig. 8a). As this is occurring, the tip is fully submerged inside the oil–water interface (Fig. 8b). During the whole interface movement phase, the extent of emulsion formation is noticeably higher when the horn’s tip is positioned closer to the interfaces center.

At 48 ms emulsion formation decreases as the interface moves past the tip (Fig. 8b). Only the lower most layer of the interface is left below the horn, decreasing the amount of oil–water interface in proximity to the cavitation area.

Due to emulsion still being produced, although on a smaller scale, the now mostly emulsion replaced water phase still increases in volume, causing the interface to continuously rise. During this its lower layer stays on the height of the horn’s tip (Fig. 8b at 79 ms), supplying both water and oil to the cavitation area and enabling further emulsion formation, while the upper layer moves upwards. A narrow emulsion stream is still slightly visible (Fig. 8b), although mostly obscured by the already formed emulsion.

A small segment of the interface is still present below the tip 103 ms after horn activation (Fig. 8b), still allowing for emulsion formation and the rise of the interface.

Fig. 8 at 150 ms represents the end stage, when all the water phase has been homogeneously replaced by the formed O/W emulsion, while most of the oil phase has been left unaffected.

#### 3.2.2 Inside the interface

**Fig. 9:**
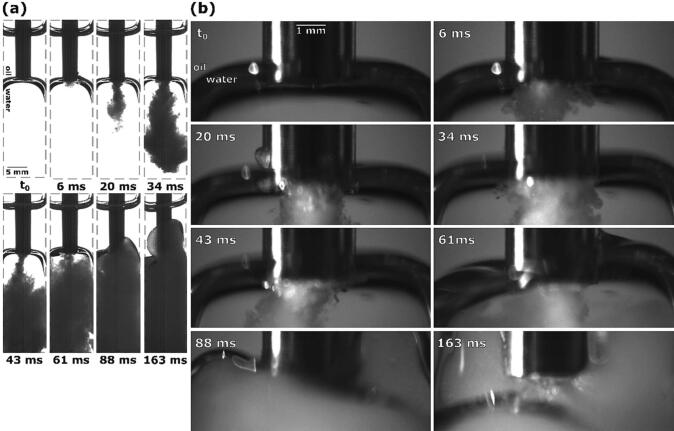
Sequence of events during ultrasonic emulsification under visible light with the ultrasonic horn positioned inside the oil–water interface: (a) observation with Photron Mini UX100, (b) observations with Photron SA-Z.

For more insight into ultrasonic horn emulsification mechanisms, we then positioned the tip inside the oil–water interface, as close to its center as possible (Fig. 9b). This induced a slight downwards pressure on the interface, causing it to become flatter in comparison to previous cases.

6 ms after horn activation an initial emulsion cone starts to form from the center of the tip (Fig. 9), in contrast to earlier cases where emulsion clouds formed at the tip’s corners. No interface deformation is visible at this time.

At 20 ms the initial emulsion cone transforms into a steady emulsion stream. At the same time we see that the whole interface becomes narrower, caused by the horn’s oscillation, which pulls the upper interface layer down and the lower interface layer up.

The next noteworthy event occurs at 34 ms, when the formed emulsion supplants most of the water phase (Fig. 9a). This change in volume causes the oil–water interface to rise higher. While enough interface is still left bellow the horn, the width of the emulsion stream increases, and more emulsion is being created.

As the lower layer of the interface mostly passes the horn’s tip, the extent of emulsion formation again reduces at 43 ms (Fig. 9).

Continuous emulsion production causes the interface to further rise, but at 61 ms its lower segment still stays at the height of the tip, while the upper part continues to move up. Consequently, the interface begins to thicken. The lower interface part being near the tip enables uninterrupted emulsion formation.

At 88 ms most of the interface has split off from the horn’s tip, leaving only a thin segment left at that height for further emulsion formation (Fig. 9b) although almost all of the water phase has been used up for emulsion production and replaced by it. As the top part rises higher, it curves outwards into the remaining oil phase.

163 ms after ultrasonic horn activation the whole water phase has been incorporated into the formed emulsion. An advantageous position of the formed emulsion, where the horn’s tip becomes visible at the end (Fig. 9b), offers us a unique insight into the end stages of emulsion formation. Here, no emulsion stream is visible and only cavitation is present underneath the tip.

#### 3.2.3 Directly below the interface

**Fig. 10:**
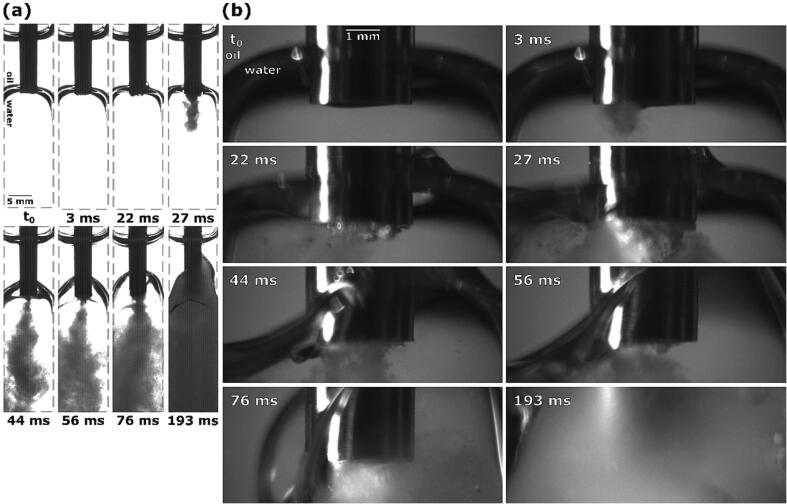
Sequence of events during ultrasonic emulsification under visible light with the ultrasonic horn positioned slightly below the oil–water interface: (a) observation with Photron Mini UX100, (b) observations with Photron SA-Z.

Further on, we positioned the tip slightly below the lowest layer of the oil–water interface (Fig. 10). Similarly to the previous cases where the tip is in close vicinity of the interface, the horn piercing caused the interface to be slightly flattened.

**Fig. 11:**
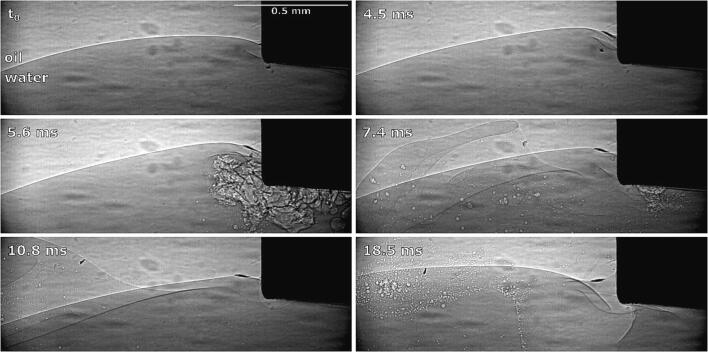
Synchrotron observations of brief emulsion formation at ultrasonic horn’s corner and downwards interface movement.

3 ms after horn activation, cavitation starts to develop and an initial emulsion cloud between the left corner and center of the horn forms (Fig. 10b). Emulsion formation might have begun at this location due to an unnoticeably small inclination of the tip, meaning the lowering interface reached that corner of the horn first (see further discussion of Fig. 11). The same was noticed in all other repetitions.

Fig. 10 at 22 ms shows that further horn movement causes the interface to split and its lower layer to be pulled down towards the tip, while parts of the upper layer start to climb up the horn. At this point cavitation begins to occur over the whole tip, continuously producing an extremely low amount of emulsion (Fig. 10b).

27 ms after horn activation, sections of the interface reach low enough for an emulsion stream to start forming (Fig. 10a). This occurs directly, without the prior formation of larger emulsion clouds.

The interface continues to split into layers more distinctively at 44 ms (Fig. 10a). During this the lower layer remains roughly at the same height as the tip, while the upper layer moves higher up and becomes pointier. A noticeable reduction in emulsion production also occurs during this time, visible by the emulsion stream being narrower immediately below the horn compared to the previous observation (Fig. 10b).

At 56 ms the lower interface section begins to split off from the horn and move downwards into the water phase (Fig. 10a). In Fig. 10b it is visible that curved segments of the interface are still present near the tip. The extent of emulsion formation remains unchanged.

As the distance between the lower interface layer and the horn increases, a new section of interface starts protruding towards the tip (Fig. 10a at 76 ms), enabling continuous and more extensive emulsion formation.

Such emulsion formation continues all the way to the end stage at 193 ms when the whole water phase has been incorporated into the O/W emulsion, homogenously replacing it.

As already mentioned (Fig. 10 at 3 ms), we noticed initial and brief emulsion cloud formation near the horn’s left corner. By synchrotron measurements (Fig. 11), we can clearly observe this.

The emulsion cloud formation is caused by the slightly uneven positioning of the horn’s tip. Emulsion from this area is spreading horizontally until the initial emulsion cloud itself quickly disappears, after which the interface further from the horn moves significantly higher, splits into several layers near the horn’s corner and curves around it.

### 3.3 Ultrasonic horn positioned lower the oil–water interface

Finally ultrasonic horn placement below the oil–water interface was observed. For these investigations we initially positioned the horn’s tip in 2 locations relative to the interface, such that the distance between them measured roughly 1.0 mm and 2.0 mm, respectively.

#### 3.3.1 1.0 mm below the interface

**Fig. 12:**
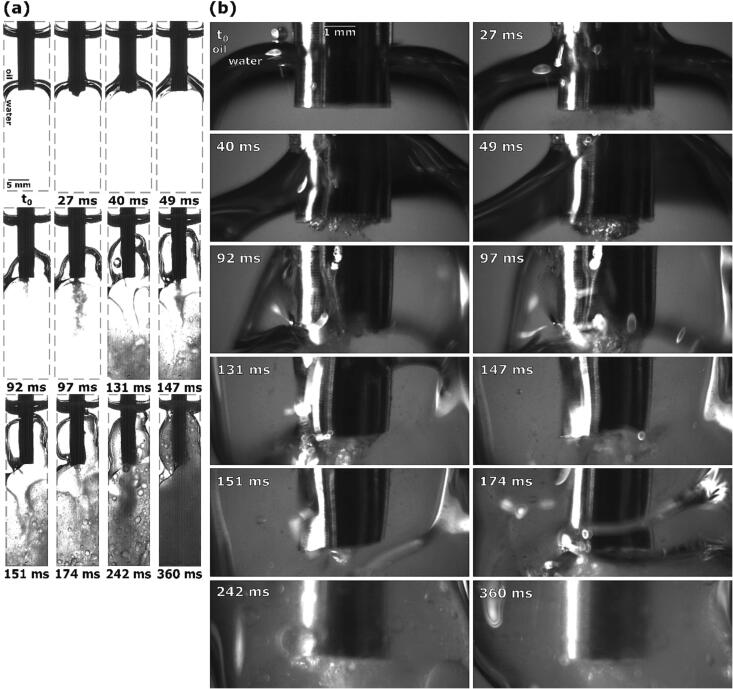
Sequence of events during ultrasonic emulsification under visible light with the ultrasonic horn positioned 1.0 mm below the oil–water interface: (a) observation with Photron Mini UX100, (b) observations with Photron SA-Z.

The ultrasonic horn’s lowest point was positioned 1.0 mm below the lowermost interface layer. What is immediately noticeable for these examples (Fig. 12) is that individual phenomena took significantly longer to occur in comparison with other ultrasonic horn placements, and as such the intervals between events are longer.

The first things to occur after horn activation, includes the upper oil–water interface moving and curving upwards at the edges of the horn, as well as small shifts of the inner interface layers. Interface curving steadily increases and at 27 ms parts of this curved layer are visibly climbing up the horn (Fig. 12a).

As the effects of the horn’s oscillation continue to escalate, the interface becomes thicker and moves further up the horn (Fig. 12 at 40 ms). This widening of the interface is more pronounced closer to the horn, as the interface’s top layers continue to climb up the horn, while its lower layers start to move downwards. The individual interface parts left and right of the horn also become straighter.

Further interface movement leads to the separation of its layers at 49 ms. The new lower interface segment appears to be much thinner than its upper counterpart. The onset of emulsion production is still not visible at this point.

As most of the interface continues to move upwards the parts that were reaching towards the tip become thinner and thus almost invisible (Fig. 12a at 92 ms). These parts simultaneously move downwards past the tip and into the water phase, while a smaller emulsion cone starts to form and pierce through them. The larger interface section that was previously visible near the horn (Fig. 12b) has now disappeared, instead only a very thin, upwards curved part behind the horn remains.

For a very brief moment at 97 ms emulsion production increases and a full emulsion stream forms (Fig. 12a). The upper interface starts to deform while parts of the interface are still visible near the tip (Fig. 12b).

By 131 ms the emulsion stream disappears but the remaining current from the horn starts to deform the interface elements inside the water phase by pushing them downwards (Fig. 12a). Although barely visible yet, it appears the interface is being ripped apart by this, and a few bigger oil droplets become visible at the lover ends of the cuvette. Fig. 12b shows how some parts of the interface are again drawn closer to the horn’s tip.

When segments of the interface at 147 ms again reach the horn’s tip, a new emulsion stream forms, with sizable parts of the upper interface layer being present near the horn and its tip (Fig. 12a). Simultaneously, the lower interface parts are still being broken apart inside the water phase, creating a coarser emulsion.

At 151 ms the emulsion stream disappears again. Such interchanging phases of emulsion formation and non-emulsion formation were present in other parallels with the same initial horn position as well, although with somewhat different frequencies compared to this particular example.

The interchanging phases of fine emulsion and larger oil bubble formation lead at 174 ms to the accumulation of both fine and coarse emulsion in the bottom half of the cuvette (Fig. 12a). Both displace unused water from the water phase, which results in the upper layers of the oil–water interface being pushed further up towards the oil-air interface.

As more emulsion is periodically forming, water from the bulk water phase is used up and more formed emulsion is beginning to accumulate further up the cuvette. Interestingly, transparent parts are visible in Fig. 12a at 242 ms near the tip, where seemingly almost no emulsion is present, but rather the formed emulsion just passes through it when moving up.

Fig. 12 at 360 ms represents the conclusion of this type of emulsion formation. The water phase becomes covered with both the fine and coarse emulsion. Larger oil droplets are still present throughout the whole water/emulsion phase, although less visible further down the cuvette.

**Fig. 13:**
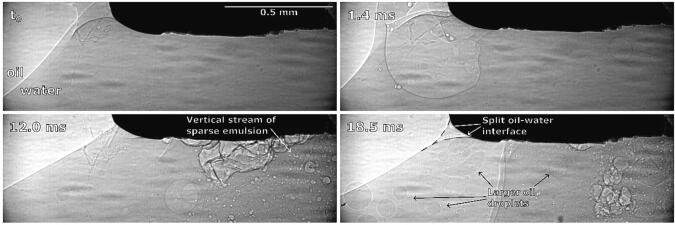
Synchrotron measurements of ultrasonic horn pushing parts of the oil phase into the water phase and subsequent emulsion formation.

Using X-Rays (Fig. 13) we observe that the ultrasonic horn will “push” larger chunks of the oil phase into the water phase (Fig. 13 at 1.4 ms) when the interface is connected to the horn’s corner from below. Horn oscillation causes the interface to slightly move downwards towards the horns corner and as it reaches low enough a split of the interface becomes visible near said corner (Fig. 13 at 18.5 ms, marked with red dashed lines). One part of this split interface is curved upwards to the side of the horn and the other part is reaching underneath it. The latter probably enables emulsion formation. However, this emulsion is sparse, scattered and consists of much larger oil droplets.

**Fig. 14:**
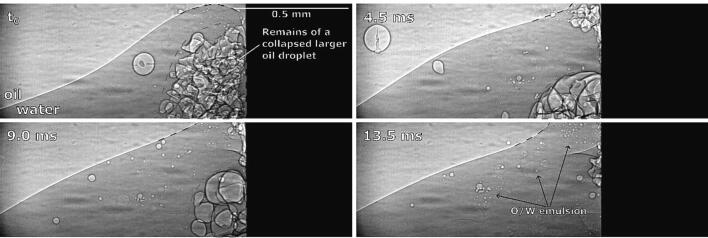
Synchrotron observations of oil–water interface climbing the ultrasonic horn’s side (the droplets circled with the red dashed lines are the remains of a collapsed larger oil droplet, observed previously in the recording, and have no connection to the discussed process itself). (For interpretation of the references to colour in this figure legend, the reader is referred to the web version of this article.)

“Climbing” of the interface can be explained by observation of emulsification under X-Rays (Fig. 14).

The climbing process is relatively slow - it continues throughout the length of observation. We can observe that parts of the visible interface segment start to reach above the field of view (Fig. 14 at 4.5 ms) in addition to this part becoming wider and stepper (Fig. 14 at 9.0 ms and 13.5 ms, marked with blue dashed lines), indicating that it moves upwards at the horns side. A small amount of emulsion is formed during this process.

#### 3.3.2 2.0 mm below the interface

**Fig. 15:**
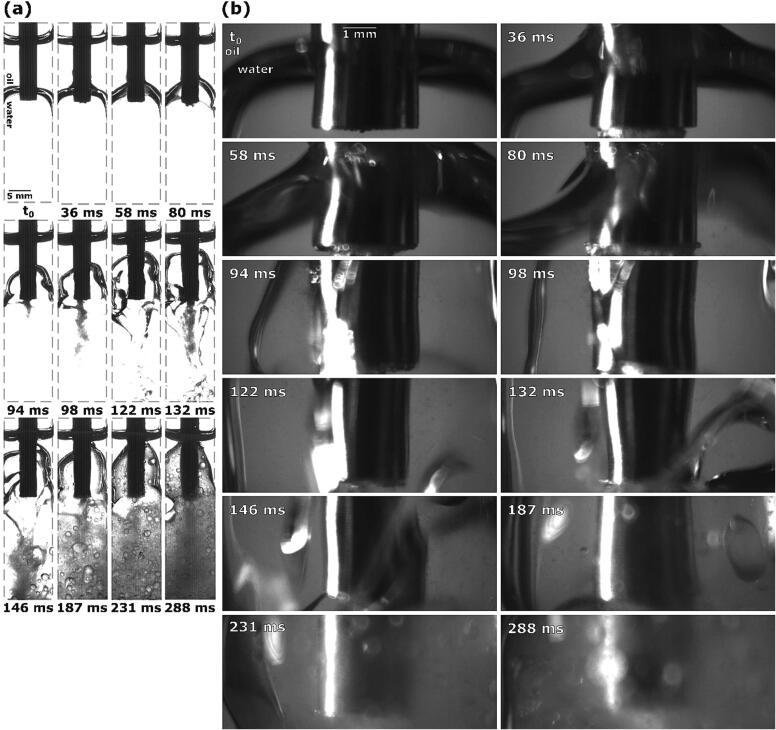
Sequence of events during ultrasonic emulsification under visible light with the ultrasonic horn positioned 2.0 mm below the oil–water interface: (a) observation with Photron Mini UX100, (b) observations with Photron SA-Z. Individual phenomena are similar to those presented on Fig. 12, mostly differing in time of appearance and appearing later due to a closer distance between horn and interface.

Individual emulsification steps here (Fig. 15) are almost identical to the ones at a smaller submergence of the horn (Section 3.3.1).

Obvious differences between them include slower interface movement in the first few phases after ultrasonic horn activation and the interfaces lower layer reaching the horn’s tip later (Fig. 15 at 80 ms, compared to Fig. 12 at 49 ms) due to the larger distance it must overcome. However, the initial emulsion streams, which form as parts of the interface connect with the horn’s tip (Fig. 15 at 98 ms), appear roughly at the same time. After this, intervals of emulsion formation and non-emulsion formation can in both examples occur with different frequencies and durations, as well as the “tearing” of the oil–water interface into larger oil droplets (i.e., formation of the coarser emulsion, Fig. 15b at 132 ms) cause small differences in the timings of events. Nevertheless, the produced emulsion is noticeably coarser than in the case when the ultrasonic horn is initially positioned above the interface (Fig. 10), with visibly larger droplets of oil mixed in-between the finer emulsion (Fig. 15b at 288 ms).

### 3.4 The influence of sonification time and initial ultrasonic horn position on emulsion size distribution

**Fig. 16:**
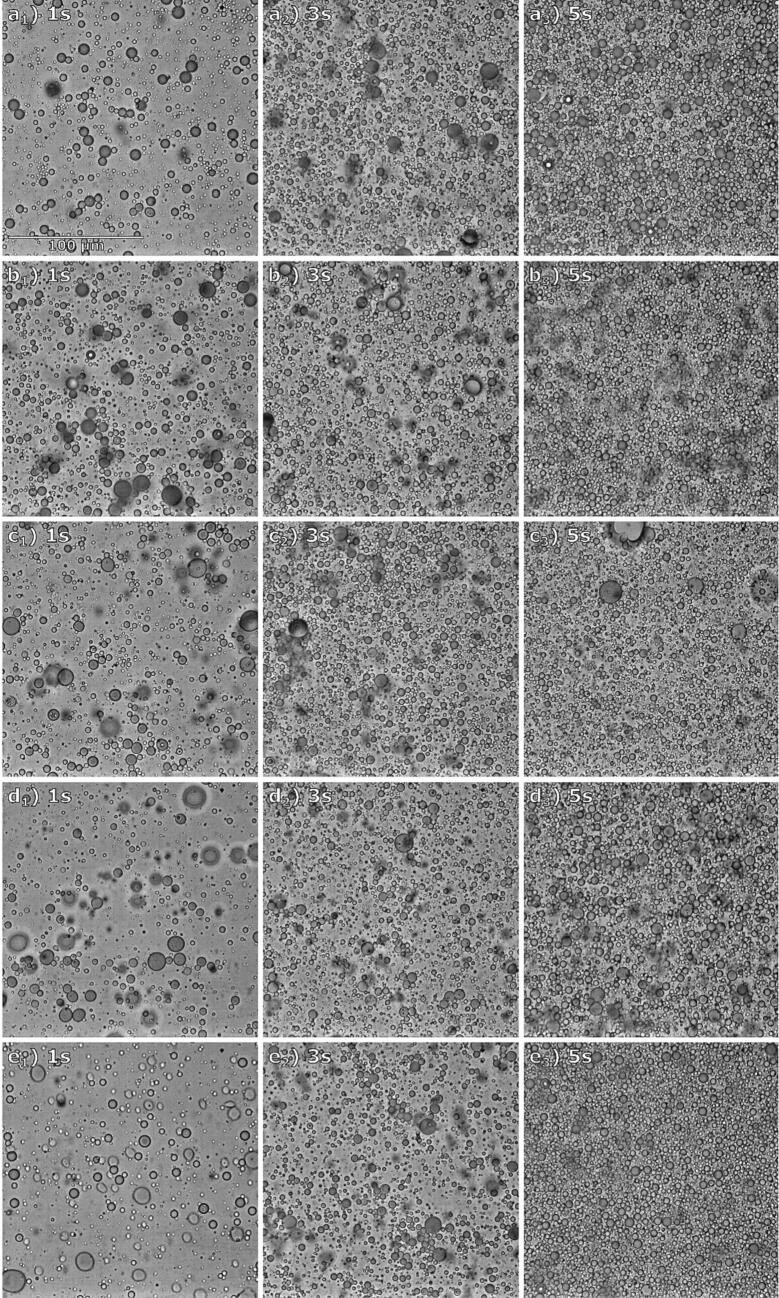
Example images of prepared emulsions after 1, 3 and 5 s of sonification with different initial horn position: (a) 2 mm and (b) 1 mm above the interface, (c) inside the interface, (d) 1 mm and (e) 2 mm below the interface. Note that these images do not represent the average emulsion drop diameter or size distribution for their respective sample.

A separate series of experiments was undertaken to determine the influence of initial ultrasonic horn placement on the size, more specifically the size distribution, of emulsion droplets after 1 to 5 s of sonification. Examples of the acquired images are presented in Fig. 16.

What is immediately obvious and was also expected is the steady increase in the number of emulsion droplets during a longer sonification time. After longer periods of sonification, the amount of leftover oil in the oil layer was also lower (results not shown), agreeing with the increased number of oil droplets inside the water phase.

**Fig. 17:**
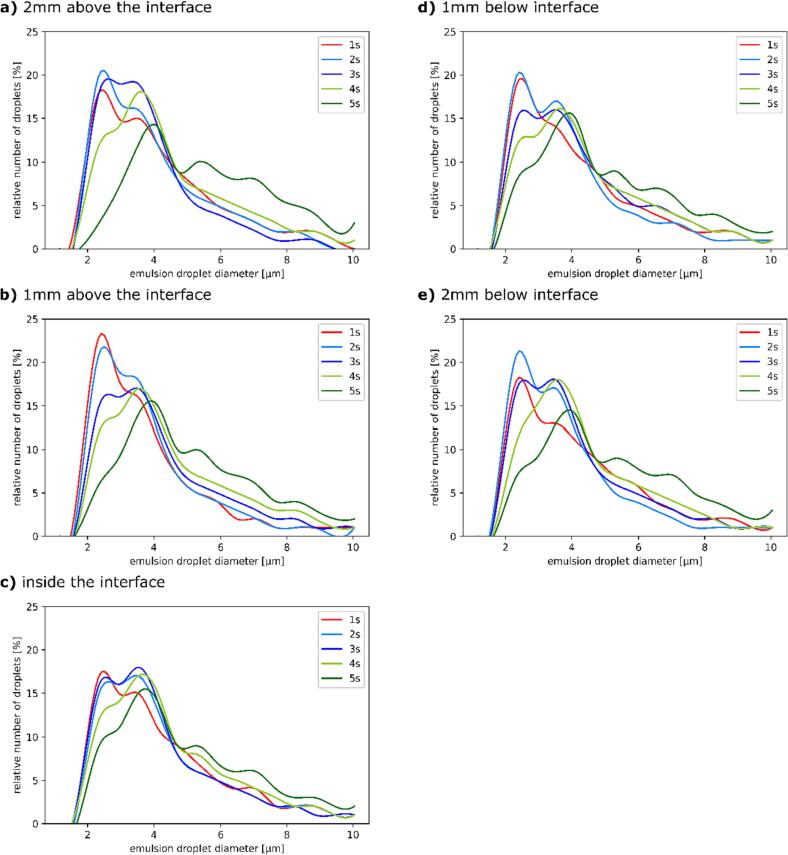
Comparison of relative number of droplets vs. emulsion droplet diameter after 1–5 s of sonification for different initial ultrasonic horn positions: (a) 2 mm and (b) 1 mm above the interface, (c) inside the interface, (d) 1 mm and (e) 2 mm below the interface.

The relative number of emulsion droplets with a certain size for a sample series was plotted against their diameters. The comparison for these results for sonification times of 1–5 s and all 5 initial horn positions are shown in Fig. 17.

With the increase in sonification time the initial peak for emulsion size distribution starts to shift towards larger emulsion droplets for all examples, either steadily lowering or shifting the first and largest peak. This increase in emulsion droplet size can be attributed to over-processing, a phenomenon caused by excess energy input during emulsification, which leads to droplet re-coalescence [18], [19]. For examples where the horn was initially placed 2 mm above or below the oil–water interface (Fig. 17a and Fig. 17e) a noticeable increase in the share of emulsion droplets with a diameter of around 2.5 µm and 3.5 µm occurs from 1 s to 2 s of sonification. Sonification for 3 s causes the peaks around 2.5 µm and 3.5 µm to even out. Longer sonification times (4 and 5 s) result in the disappearance of the first peak and slight shift of the second towards 4 µm. After 5 s of sonification the samples where the horn was initially placed 2 mm from the oil–water interface exhibit similar trends of relative numbers of droplets for emulsion sized between 5 µm and 10 µm, with slight peaks around 5.5 µm and 7 µm.

Analogous to the droplet size distribution of examples where the initial distance between the horn and oil–water interface was 2 mm, the examples where this distance was 1 mm (Fig. 17b and Fig. 17d) also behave similar to one another. Here the first peak of emulsion droplets (around 2.5 µm) stays roughly the same when increasing the sonification time from 1 s to 2 s. However, a more pronounced increase of the share of emulsion droplets with the diameter around 3.5 µm occurs during the same time period. Further increase in sonification time to 3, 4 and 5 s causes the peak around droplets 2.5 µm in diameter to steadily decrease, while the peak around 3.5 µm slightly shifts towards 4 µm but the relative number of emulsion droplets at this size stays roughly the same. The trend for relative number of emulsion droplets with diameters between 5 µm and 10 µm after 5 s of sonification is also similar as 3 peaks occur for droplets 5.5 µm, 7 µm and 8.5 µm in diameter.

The samples where the horn was initially placed inside the oil–water interface (Fig. 17c) exhibits the lowest share of emulsion droplets with a diameter around 2.5 µm after 1 s of sonification. Comparable to other examples a new peak starts forming for droplets around 3.5 µm in diameter with increased sonification time while the initial peak around 2.5 µm steadily disappears. While initially increasing, the newly formed peak around 3.5 µm starts to decrease after 4 s and even more after 5 s of sonification.

**Fig. 18:**
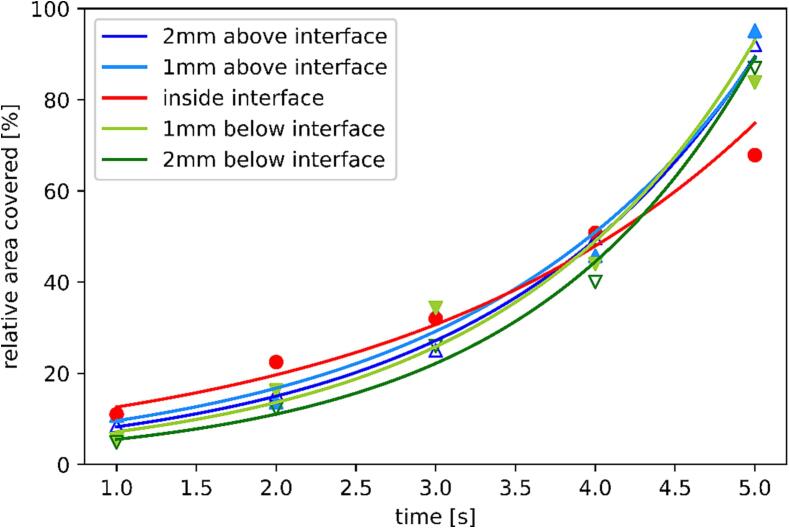
Relative area covered by emulsion droplets vs. time of sonification for different initial horn positions: 2 mm (dark blue Δ) and 1 mm (light blue ▲) above, inside (red ●), 1 mm (light green ▾) and 2 mm (dark green ∇) below the interface. The curves represent the exponential functions fitted to the dataset for emulsion prepared with different horn positions: 2 mm (dark blue) and 1 mm (light blue) above, inside (red), 1 mm (light green) and 2 mm (dark green) below the oil–water interface. (For interpretation of the references to colour in this figure legend, the reader is referred to the web version of this article.)

The analysis of microscopy images of emulsions described in the beginning of Section 3.4 returned the number of detected emulsion droplets and their diameters. This data was further used to calculate the area emulsion droplets occupied on these images for each case (specific horn position and sonification time). These emulsion areas were divided by the whole area of the images (number of images for each case multiplied by their area of 189.1 × 189.1 µm), resulting in the calculation of relative areas occupied by emulsion for each case. These results are presented in Table 1 and Fig. 18 (as triangle and circular symbols).

**Table 1:** Calculations of relative area covered by emulsion droplets for each initial horn position after 1–5 s of sonification.

	relative area covered by emulsion [%]				
	1 s	2 s	3 s	4 s	5 s
2 mm above interface	8.40	15.47	24.99	49.69	92.02
1 mm above interface	10.75	13.69	33.23	45.77	95.14
inside interface	11.01	22.46	32.01	50.82	67.86
1 mm below interface	5.61	16.22	34.36	43.99	83.78
2 mm below interface	4.84	12.23	25.98	40.06	86.96

Placing the ultrasonic horn inside the oil–water interface results in the formation of substantially more emulsion (around 22 %) after 2 s of sonification compared to other horn positions (around 12–16 %). However, these differences fade at 3 and 4 s of sonification and the inverse occurs at 5 s, when the relative amount of produced emulsion by placing the horn inside the interface is around 68 % (compared to around 84–95 % for other positions). The values of relative area covered by formed emulsion were generally lower when the horn was submerged below the oil–water interface compared to when the horn was position above it.

To better present/quantify the trend of increasing emulsion amount we fitted exponential functions to the results of relative area covered by emulsion from Table 1. These fitted functions are presented as curves on Fig. 18. Both a linear and an exponential function could be fitted to the results for emulsion production with the horn initially inside the oil–water interface (Fig. 18, red circles) with an almost identical coefficient of determination (R^2^). We chose to fit it to the latter since exponential functions were also fitted to all other datasets. The coefficients of this statistical analysis are shown in Table 2.

**Table 2:** Results of fitting exponential functions with coefficients c_1_ and c_2_ to the relative area covered by emulsions produced with different initial ultrasonic horn placement and their coefficients of determination (R^2^).

$\left(A_{\mathrm{em}}=c_{1}*e^{c_{2}}\right)$	c_1_	c_2_	R^2^
2 mm above interface	4.56	0.60	1.00
1 mm above interface	5.50	0.56	0.98
inside interface	8.07	0.45	0.98
1 mm below interface	3.78	0.64	0.98
2 mm below interface	2.74	0.70	0.99

The 1st coefficient of the fitted exponential functions (c_1_) increases in value with the decrease in distance between the horn and oil–water interface, reaching its maximum when the horn was located inside the interface. When submerging the horn below the interface, this trend continues as the values of c_1_ decrease with the increase in distance between horn and interface. The complete opposite occurs for the 2nd coefficient (c_2_), which reaches its minimum when the horn is placed inside the interface and increases in value when the distance between horn and interface increase (in both directions).

## 4 Discussion

We found that in all above-described emulsion formation examples the oil–water interface splits into multiple parts, either before or after the onset of the emulsion stream. In all these cases, some parts of the split interface travel up the ultrasonic horn. When the horn’s starting position is inside the oil phase (Fig. 3, Fig. 7, Fig. 8) or inside the oil–water interface itself (Fig. 9), lower segments of the interface stay level with the horn’s tip during emulsion formation, while parts of the interface move down past the tip and into the water phase in cases where the horn was initially placed below the interface (Fig. 10, Fig. 12, Fig. 15).

Common to all provided examples is also the fact that the only formed emulsion is O/W emulsion and never W/O, regardless of the horn’s starting position relative to the interface. This is evident due to the emulsion only being present inside the water phase, meanwhile most of the oil is left unchanged after finished sonification. The provided results additionally indicate that once the horn is positioned further away from the oil–water interface, be it above or below, the exact distance between them doesn’t influence general emulsification mechanisms, only the time at which they occur.

In the example where the ultrasonic horn is positioned highest above the oil–water interface (Section 3.1.1) the extent of the emulsion stream, once fully formed, never decreases, unlike in the example from Section 3.1.2. This is because by the time the main interface segments reach the horn’s tip, the majority of emulsion has already been produced, meaning that amounts of additionally formed emulsion will not be enough to sufficiently elevate the interface above the horn’s tip, thus not decreasing their contact and diminishing emulsion production.

We also observed during cases with ultrasonic horn placement above the interface the deformation of said interface (Fig. 3 at 13 ms and Fig. 7 at 10 ms) and the formation of peaks from its top layer towards the horn. One can be seen inclined to the left in Fig. 7 at 10 ms and two rotating outwards in Fig. 3 at 16 ms. These rotating peaks are consistent with circular fluid flow present at the horn’s corners, confirmed through Particle Image Velocimetry (PIV) experiments by Lebon et al. [20]. According to their findings, circular currents are oriented clockwise at the right corner and counterclockwise at the left corner. The conical interface protrusion first noticeable in Fig. 3 at 13 ms and Fig. 7 at 10 ms could be caused by shock wave emissions from imploding bubbles. According to Khavari et al. [21] these shock waves influence greatly the pressure distribution in the liquid and the pressure is highest directly underneath the ultrasonic horn. Since most of the formed cavitation bubbles are located at its center, where they function as energy absorbers, the resulting shock waves are weaker in the center and stronger near the horn’s edges, from where they spread vertically and deform the interface. Additionally,0 the presence of such shock waves could explain the formed vertical emulsion streams seen in Fig. 3 at 20 ms and Fig. 7 at 14 ms after the circular interface protrusions reach the horn.

The number of individual steps for emulsion formation in examples where the horn is initially positioned inside the oil–water interface (Section 3.2.2) is lower than for examples where the horn is slightly elevated above the interface (Section 3.2.1). The onset of emulsion generation in the former is most likely not proceeded by greater shifts in interface position since it is already ideally positioned for emulsion formation, whereas in the latter example the interface must first move upwards. Similarly in examples where the interface initially lies lower (Section 3.2.3), noticeable downwards shift of the interface must occur before emulsion production can begin, although less complex than in examples above the interface (Section 3.2.1).

Interestingly, the visual observations from Section 3.3 (the ultrasonic horn positioned below the oil–water interface) indicate that such a horn position is less suitable for fine emulsion production, as the emulsion formed in these examples is visibly coarser, with noticeably larger oil drops inside the water phase mixed with finer emulsion. They are the result of the ultrasonic horn induced flow “ripping” apart lower parts of the interface inside the water phase, as seen in Fig. 12a at 130 ms and Fig. 15a at 122 ms. These drops, being much larger than fine emulsion droplets, are outside the detection range of the Python algorithm used. This means that even though more oil has been used up in examples when the horn was submerged inside the water phase (evident by the lower amounts of leftover oil, Fig. 12a at 360 ms and Fig. 15a at 288 ms) this has no influence on droplet size distribution for fine emulsion (Fig. 17d and Fig. 17e).

After the increase of sonification time from 1 to 2 s the increased ultrasonic power probably breaks down the aforementioned larger oil drops (seen in Fig. 15a from 146 ms to 288 ms) into finer emulsion. This would explain the largest initial increase in share of droplets with smaller diameters (Fig. 17e around 2.5 µm and 3.5 µm) detected when the horn was placed 2 mm below the interface compared to when the horn was positioned 2 mm above it (Fig. 17a). Further increase in sonification time shifts the droplet sizes to higher values, probably again due to over-processing [18], [19]. This could potentially be mitigated by the utilization of pulsed-mode sonification, where silent periods in between pulses supposedly increase transient cavitation intensity at lower energy consumption [22]. However, this would need to be carefully examined in regard to the ultrasonic emulsification process.

As described in Section 3.4, longer sonification times seem to affect emulsion droplet size distribution in a similar way when the distance from the horn and oil–water interface is equal (Fig. 17a and Fig. 17e, Fig. 17b and Fig. 17d), whereas placing the horn inside the interface (Fig. 17c) results in completely unique behavior of droplet size distribution. This indicates that while the emulsion formation process is influenced by the horn position relative to the interface (i.e., above or below it), the emulsion droplet size distribution is dependent on the distance between horn and interface. The share of oil in O/W emulsion prepared with ultrasonic horn appears to increase exponentially for all horn positions (Fig. 18). This increase is slightly slower when the horn is initially placed inside the interface (Fig. 18, red curve).

**Fig. 19:**
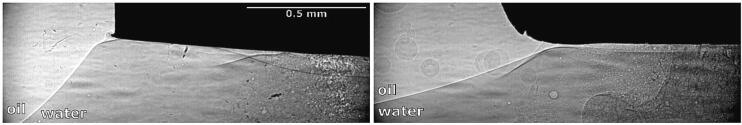
Synchrotron observations of emulsion stream formation from a thin oil layer underneath the ultrasonic horn (frames taken from 2 different examples).

From the above provided results we presume that emulsification mechanisms with ultrasonic horn follow 2 pathways: when the horn is initially positioned above the oil–water interface or below it. In both cases the interface needs to first reach the tip where cavitation is present, either by moving and deforming up or down. This occurs due to the induced flow fields, which are according to Lebon et al. [20] oriented up towards the horn’s tip and form vortices at the corners, and due to vertically moving shock waves, caused by bubble collapse [21]. Emulsion production in the form of a fine emulsion stream then occurs when a big enough part of the oil–water interface is in close proximity to the ultrasonic horn’s tip, visible for example in Fig. 9 at 6 ms, Fig. 10 at 27 ms, Fig. 3 at 37 ms and Fig. 15 at 132 ms. This leads us to conclude that for an emulsion stream to form, the layout of individual phases underneath the horn (from highest to lowest) must be as followed: a thin oil layer, the oil–water interface, and the bulk water phase (Fig. 19).

Comparing the images for the cases when the tip was closest to the interface - Fig. 8 at 36 ms, Fig. 9 at 34 ms and Fig. 10 at 27 ms, one can see that the widest and therefore strongest emulsion stream forms when the horn’s tip is positioned inside the interface (Fig. 9). This again points to the conclusion that a thin layer of oil is required below the horn for the formation of a strong emulsion stream.

**Fig. 20:**
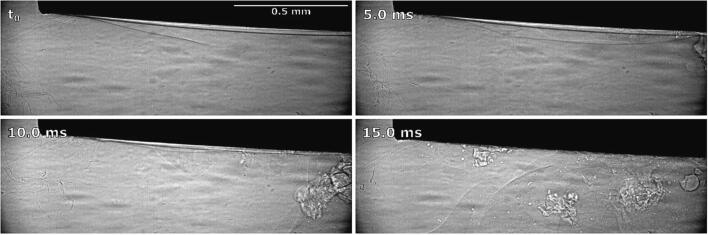
Synchrotron observation of emulsion formation from thin oil layer underneath ultrasonic horn with the gradual disappearance of aforementioned oil layer.

This would explain why in the first stages of emulsion production in examples where the horn is initially placed above the oil–water interface (Fig. 3 at 20 ms and Fig. 7 at 14 ms), oblique emulsion streams form, since the oil–water interface is positioned diagonally. The parts of the oil–water interface that connect the main interface segments to the ultrasonic horn enable a continuous supply of oil towards the horn, thus enabling the continuous formation of emulsion. Otherwise, the thin oil layer beneath the horn is used up during emulsion production, as seen in Fig. 20, causing emulsion formation to cease. The newformed emulsion displaces the older emulsion downwards, creating the emulsion stream, the width of which is dependent on the amount of new emulsion being formed.

**Fig. 21:**
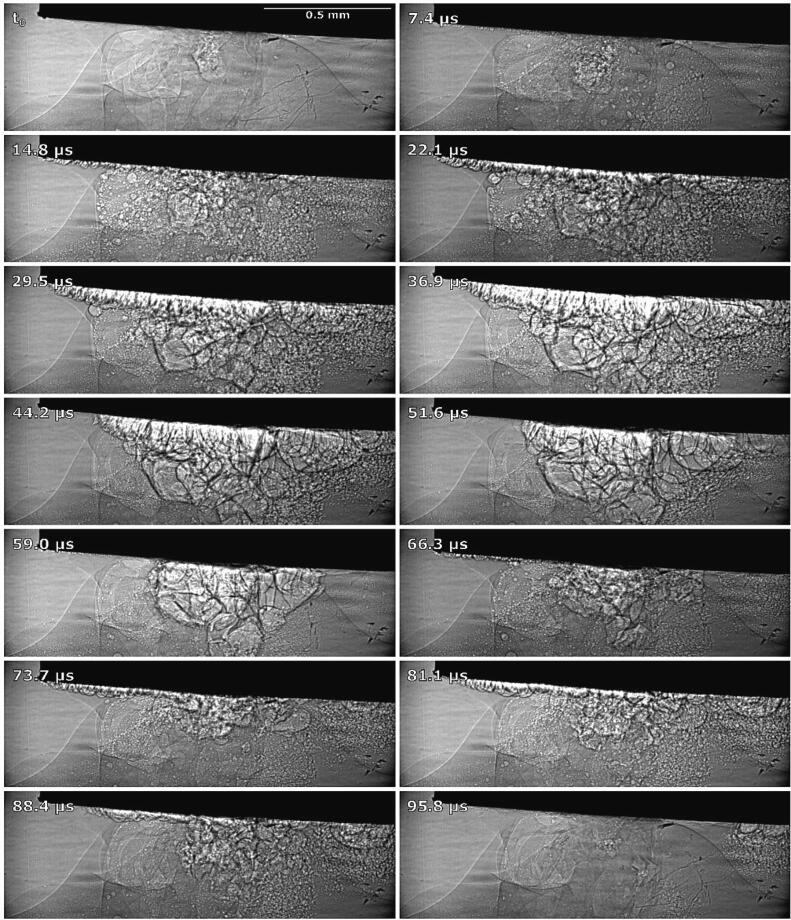
Synchrotron observations of emulsion stream formation: creation of coarser emulsion, cavitation bubble growth and dispersion of larger oil drops into finer emulsion during a 100 µs interval or 2 “oscillations” of the ultrasonic horn.

We theorize that the oscillating ultrasonic horn pushes relatively large fragments of oil from the thin oil layer into the water phase (as seen during synchrotron measurements in Fig. 21). This creates a coarse emulsion with larger droplets of oil inside the water phase, which are then immediately broken up and dispersed by cavitation, resulting in a fine O/W emulsion. Since cavitation bubbles near a liquid–liquid interface always collapse towards the interface if they grow inside the lighter phase (i.e., the oil phase) and collapse away from it if they grow inside the denser phase, this would imply that cavitation bubbles growing inside the large oil drops collapse outwards, ripping the drops apart and dispersing the oil as a fine O/W emulsion [12].

**Fig. 22:**
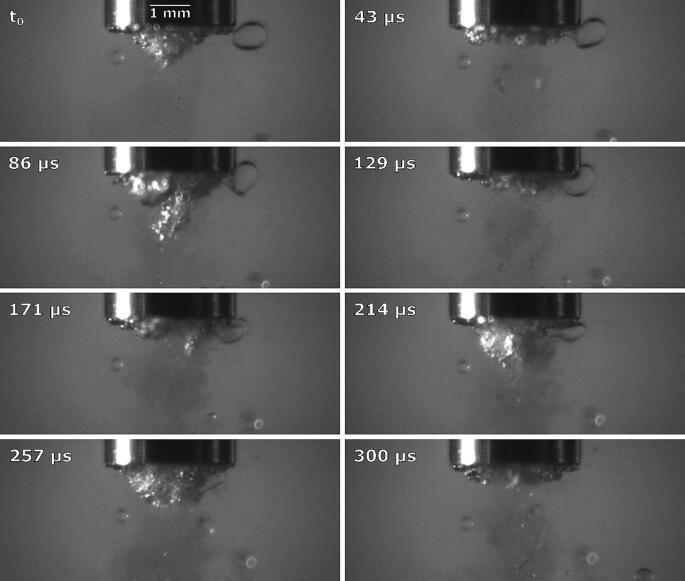
Photron SA-Z observations under visible light of a large oil droplet (near the tip’s right corner) collapsing into fine emulsion due to cavitation underneath the ultrasonic horn.

The above explained phenomena are possibly the reason why even during the emulsification process no W/O emulsion is visibly created, since water is never dispersed into the larger oil droplets or into the bulk oil phase by cavitation. Furthermore, we observed the collapse of larger oil droplets, formed due to the ultrasonic horn’s initial position inside the water phase, in the cavitation area underneath the horn, where they were transformed into fine emulsion and combine with the emulsion stream (Fig. 22).

## 5 Conclusions

The main finding that we conclude from the here presented research is that, for emulsion or rather an emulsion stream to form, both the water and oil phase need to be present in very close proximity to the ultrasonic horn’s tip, i.e., a large enough oil layer below the horn needs to be followed by the oil–water interface and then the bulk water phase. The horn’s downwards movement pushes parts of the oil phase through the interface and into the water. As the horn cycles up again, the drop in pressure causes cavitation bubbles to grow, which also grow inside the larger oil droplets, collapsing towards the interface that forms their edges, ripping them apart from within and dispersing the oil as a fine O/W emulsion. With horn oscillation these cycles continue, steadily forming new emulsion and thus creating an emulsion stream.

An initial position of the ultrasonic horn below the oil–water interface is less suitable for fine emulsion formation. The hereby produced emulsion contains numerous larger oil droplets mixed with the finer emulsion. These bigger droplets are the result of horn induced fluid flow ripping apart segments of the oil–water interface deeper inside the water phase, where no cavitation is present to disperse the coarser emulsion into a finer one. Initial horn placement inside the interface also decreases the speed at which the amount of oil in the O/W emulsion increases. The changes in droplet size distribution with increased sonification time are, however, not influenced by horn position but rather by the distance between horn and interface.

In all discussed examples, the only formed emulsion was O/W emulsion, evident by only the water phase becoming distinctly and homogenously cloudier, while the oil layer, although reduced in volume, stays visually unchanged.

The present study represents a steppingstone from purely academic investigations of cavitation driven emulsification (single bubbles, single droplets) to a more applied case – ultrasonically driven cavitation in a container filled by water and oil.

Knowledge gained by these detailed observations can of course be applied to optimization of processes of ultrasonic emulsification but also in a wider scope – for example in continuous flow emulsifiers.

With this we hope to advance the knowledge of emulsification from the fundamentals (single or few cavitation bubbles) to applicable bulk emulsification. Based on the nature of our findings, we expect the here described requirements for emulsion formation to apply not only to large-scale ultrasonic emulsification, but also to cavitation emulsification in general. For example to a more complex technique of hydrodynamic cavitation, which is possibly more suitable for large-scale industrial emulsion production.

## Declaration of Competing Interest

The authors declare that they have no known competing financial interests or personal relationships that could have appeared to influence the work reported in this paper.
